# Transcriptomics Reveals Fast Changes in Salicylate and Jasmonate Signaling Pathways in Shoots of Carbonate-Tolerant *Arabidopsis thaliana* under Bicarbonate Exposure

**DOI:** 10.3390/ijms22031226

**Published:** 2021-01-27

**Authors:** Laura Pérez-Martín, Silvia Busoms, Roser Tolrà, Charlotte Poschenrieder

**Affiliations:** Plant Physiology Laboratory, Bioscience Faculty, Universitat Autònoma de Barcelona, C/de la Vall Moronta s/n, E-08193 Bellaterra, Spain; laura.perez.martin@uab.cat (L.P.-M.); silvia.busoms@uab.cat (S.B.); roser.tolra@uab.cat (R.T.)

**Keywords:** transcriptomic, *Arabidopsis thaliana*, alkaline stress, glucosinolate, salicylate

## Abstract

High bicarbonate concentrations of calcareous soils with high pH can affect crop performance due to different constraints. Among these, Fe deficiency has mostly been studied. The ability to mobilize sparingly soluble Fe is a key factor for tolerance. Here, a comparative transcriptomic analysis was performed with two naturally selected *Arabidopsis thaliana* demes, the carbonate-tolerant A1_(c+)_ and the sensitive T6_(c−)_. Analyses of plants exposed to either pH stress alone (pH 5.9 vs. pH 8.3) or to alkalinity caused by 10 mM NaHCO_3_ (pH 8.3) confirmed better growth and nutrient homeostasis of A1_(c+)_ under alkaline conditions. RNA-sequencing (RNA-seq) revealed that bicarbonate quickly (3 h) induced Fe deficiency-related genes in T6_(c−)_ leaves. Contrastingly, in A1_(c+)_, initial changes concerned receptor-like proteins (RLP), jasmonate (JA) and salicylate (SA) pathways, methionine-derived glucosinolates (GS), sulfur starvation, starch degradation, and cell cycle. Our results suggest that leaves of carbonate-tolerant plants do not sense iron deficiency as fast as sensitive ones. This is in line with a more efficient Fe translocation to aerial parts. In A1_(c+)_ leaves, the activation of other genes related to stress perception, signal transduction, GS, sulfur acquisition, and cell cycle precedes the induction of iron homeostasis mechanisms yielding an efficient response to bicarbonate stress.

## 1. Introduction

Several constraints can affect the growth, physiology, and metabolism of agricultural crops cultivated on calcareous/alkaline soils of arid and semi-arid regions over the world. The main anions present excessively in alkaline soils are HCO_3_^−^ and CO_3_^2−^ [1]. The pH values of most calcareous soils are within the range of 7.5 to 8.5 [2], and bicarbonate concentrations are found between 5–35 mmol L^−1^. Soil carbonates act as pH buffers and play an important role in rhizosphere processes such as nutrient availability for plants. The high pH surrounding the plant roots can disrupt the membrane potential and inhibit the absorption of essential ions [3]. Moreover, high pH lowers the availability of nitrogen (N); phosphorous (P); and micronutrients such as iron (Fe), zinc (Zn), and manganese (Mn), producing nutrient deficiencies in sensitive plants [4].

Among these nutrient deficiencies induced under alkaline soil conditions, Fe has received most attention. Although Fe is one of the most abundant elements in the earth’s crust, under basic pH conditions, it is sparingly available to plants. Iron, among other essential micronutrients, is required for multiple metabolic processes, and most crops are highly sensitive to low iron availability [5]. This fact has prompted many efforts on unrevealing the mechanisms of Fe uptake, transport, and regulation, and the tolerance to Fe deficiency [6,7,8]. Iron scarcity is a relevant consequence of high pH soils, but not the only one. Deficiency of Zn, P, N, and/or Mg are common problems for crop plants on calcareous soils.

Several studies have shown that plants perceive and respond in different ways to high pH, high HCO_3_^−^, or iron deficiency [9], and the mechanisms of carbonate tolerance remain largely unknown. Alkaline pH per se can affect root growth by inhibiting cell division and elongation [10]; ethylene modulates this effect increasing the expression of *AUX1* and of genes responsible for auxin synthesis [11]. Crosstalk between jasmonate and ethylene in response to alkalinity stress has been reported [12,13]. One way for plants to adapt to high pH is to accumulate small metabolites with buffering function, especially organic acids, for adjusting the internal pH value. However, the adjusting of external and internal pH is a process that consumes energy and reduces plant growth simultaneously [10].

Bicarbonate imposes further stress on plant performance. Common features are morpho-anatomical changes in roots; disturbance of water relations; reduction of total photosynthetic pigments and photosynthetic activity; accumulation of osmo-protectants, soluble sugars, and organic acids; and activation of the biosynthesis of antioxidant enzymes [12,14]. At the molecular level, several anion transporters and gene families such as *ALMT, NRT/POT*, and *SLAHs* are related to bicarbonate response in *Glycine max* [15,16]. The overexpression of the anion transporters *GsBOR1* (boron efflux transporter) and *GsSLAH3* (efflux of uncharacterized inorganic or organic anions) are also involved in HCO_3_^−^ tolerance. Transcriptomic experiments under alkaline stress in *Tamarix hispida*, *Ziziphus acidojujuba*, and *Glycine max* identified gene families and transcription factors (TFs) involved in NaHCO_3_ stress responses. These genes can be clustered in sensing and signal transduction pathways as calmodulin-like proteins, serine/threonine protein kinases, and cysteine-rich receptor-like protein kinases. P2/ERF, HD-ZIP, bHLH, MYB, WRKY, NAC, C2H2, HB, and TIFY are the main TFs [17,18,19]. Proteomic studies performed in *Solanum lycopersicum* comparing saline and alkaline stress further revealed downregulated proteins accounting for metabolism, energy conversion, and novel upregulated proteins involved in signaling or transport [20].

Nonetheless, the current understanding of plant response to bicarbonate stress is limited. Most studies have been performed on roots of crop species such as *Glycine max* or *Oryza sativa* [21,22]. Less information is available regarding the molecular-genetic mechanisms underlying naturally selected tolerance and fast bicarbonate responses at the gene expression level in shoots. The large genotypic and phenotypic diversity of natural populations of the genetic model plant *Arabidopsis thaliana* provides an ideal experimental scenario for exploring such mechanisms. In previous studies, we identified two contrasting demes of *A. thaliana* with differential tolerance to moderate soil alkalinity: the sensitive deme T6_(c−)_ and the tolerant A1_(c+)_. The better performance of A1_(c+)_ under iron deficiency conditions was mainly attributed to enhanced root release of coumarin-type phenolics [23]. However, there is increasing evidence for both quick shoot responses and shoot to root signaling under iron deficiency or bicarbonate stress [24,25].

The aim of this study was to characterize the differences in initial shoot responses of naturally selected *A. thaliana* demes differing in tolerance to carbonate soils. To clearly discriminate between the initial responses to high pH per se from those caused by bicarbonate, one of the most growth-limiting factors on alkaline soils, we submitted seedlings to control conditions (pH 5.9), high pH provided by 1,3-bis[tris(hydroxymethyl)methylamino]propane (BTP) buffer (pH 8.3), and alkaline conditions achieved by supplying bicarbonate (10 mM NaHCO_3_, pH 8.3). For abbreviation, pH 5.9, pH 8.3, and bic are respectively used to describe these treatments. Primary gene responses were analyzed after 3 h of exposure, while a second analysis was performed after a medium-term treatment of 48 h. Differential expression of genes and their gene ontologies were compared between demes, treatments, and time points, giving a detailed picture of the short- and longer-term responses to pH 8.3 and bic stress of *A. thaliana* plants differing in adaptation to carbonated soil.

## 2. Results and Discussion

### 2.1. Fitness Characterization of A. thaliana Plants under High pH and Bicarbonate Stress

**Figure 1:**
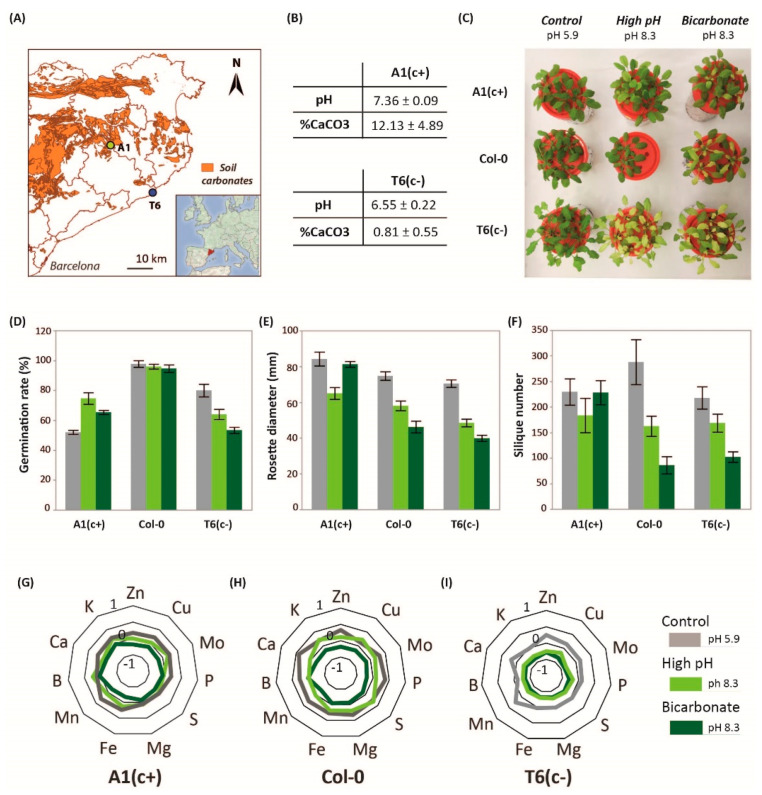
Location, soil parameters, physiological characterization, and plant nutrition. (**A**) Location of *Arabidopsis thaliana* natural populations A1_(c+)_ and T6_(c−)_ in a soil map of Catalonia. Orange areas represent calcareous soils and white areas non-calcareous soils. (**B**) Soil carbonate and pH levels (means ± Standard Error; *n* = 9) in the natural habitat of A1_(c+)_ and T6_(c−)_ populations. (**C**) Picture of 30-day-old Col-0, A1_(c+)_, and T6_(c−)_ plants under different alkaline treatments. Germination rate (**D**), rosette diameter (**E**), and silique number (**F**) of A1_(c+)_, T6_(c−)_, and Col-0 plants submitted to control (pH 5.9, grey bars), high pH (pH 8.3, light green bars), or bic (pH 8.3 with 10 mM of NaHCO_3_, dark green bars) in plates or hydroponic culture. Values are means ± SE; *n* = 45, *n* = 12, *n* = 9, respectively; letters indicate significant differences (*p* < 0.05, Tukey’s honestly significant difference HSD) per deme. (**G**–**I**) Standardized leaf mineral content of four plants per deme grown under pH 5.9 (grey lines), pH 8.3 (light green lines), or bic (dark green lines) for 2 weeks.

In previous studies, we identified two contrasting demes of *A. thaliana* with differential tolerance to moderate soil alkalinity: the sensitive population T6_(c−)_ and the tolerant A1_(c+)_ [23]. A1_(c+)_ is native to areas near to calcareous soils, and soil CaCO_3_ content and pH in its habitat are higher than in native soils of T6_(c−)_, which are derived from silica parental rock (Figure 1A,B).

In order to ascertain the bic concentration that efficiently discriminates both demes under hydroponic conditions, we performed preliminary studies using a concentration range from 0 to 20 mM NaHCO_3_. Rosette diameter and root length growth of both demes was monitored for 5 weeks (Figure S1A,B, Dataset S1). Concentrations higher than 10 mM caused high mortality before ending the life cycle. This contrasts with more carbonate-tolerant crop plants such as *Glycine max*, where 50 mM NaHCO_3_ was established as a mild stress situation avoiding high mortalities [17]. According to our results, a concentration of 10 mM NaHCO_3_ was selected as an adequate discriminatory treatment in all subsequent experiments.

The ability to germinate, i.e., root emergence, is a prerequisite for efficient plant establishment on problem soils, and seed germination rate under stress conditions has frequently been used to assess plant tolerance to soil constraints [26]. Here, seed germination tests were performed, comparing the contrasting natural demes with the model accession Col-0. Seed germination tests revealed, for all accessions, a delay in germination in the high-pH treatments (pH 8.3 and bic) in comparison to pH 5.9. However, 10 days after sowing the germination rate was hardly affected in Col 0. A1_(c+)_ seeds showed better germination rates under the high pH treatments than under pH 5.9. In contrast, both alkaline stresses reduced the germination rate of T6_(c−)_ (Figure 1D). Accordingly, A1_(c+)_ and Col-0 are more tolerant to alkaline conditions than T6_(c−)_. However, seed germination does not guarantee further growth, survival, and reproductive fitness under alkaline stress [27]

Hydroponic studies using solutions with control pH (pH 5.9), high pH (pH 8.3), and bic (10 mM NaHCO_3_, pH 8.3) confirmed the higher tolerance of A1_(c+)_ to both pH 8.3 and bic. Col-0 exhibited an intermediate response, while T6_(c−)_ was most sensitive. Both alkaline treatments, pH 8.3 and bic, induced severe chlorosis in leaves of T6_(c−)_ (Figure 1C). Bic treatment also caused stronger chlorosis symptoms in Col-0 than in A1_(c+)_, while pH 8.3 apparently had little effect in both accessions.

Rosette diameter data measured 15 days after the start of treatments and number of siliques further revealed alkaline sensitivity of both T6(_c−_) and Col-0 and confirmed tolerance of A1(_c+_) (Figure 1E,F, Dataset S1). Interestingly, while in the sensitive accessions bic was more stressful than pH 8.3, the alkaline-tolerant A1_(c+)_ performed better under bic than under pH 8.3 (Figure 1F). The observation that in sensitive plants bic is more stressful than pH 8.3 is in line with the inhibitory effects of bic on essential metabolic processes in plants [28]. Especially affected by bicarbonate are mechanisms activated under Fe deficiency conditions such as riboflavin biosynthesis genes and *NRAMP1* in roots or *bHLH38* and *IRT1* in shoots, as shown in melon plants exposed to either Fe deficiency alone or in combination with bicarbonate [29].

### 2.2. Nutrient Content Profile

Differences in tolerance to pH 8.3 and bic treatments were also reflected in the plants’ ionomic profiles. Nutrient concentrations in Col-0 were more affected by bic than by pH 8.3. In general, the tolerant deme A1_(c+)_ maintained ion homeostasis better than the sensitive plants (Figure 1G–I). Moreover, maintenance of ion homeostasis in A1_(c+)_ was better under the bic treatment than under pH 8.3. Only P, Ca, and Mn concentrations were significantly reduced by bic in all demes. Statistical significance of treatment vs. genotype interactions (*p*-value < 0.05) are shown in Dataset S1. However, all tissue concentrations in A1_(c+)_ exposed to bic remained above the deficiency thresholds for Brassicaceae species [30]. Especially relevant is the ability of A1_(c+)_ to maintain leaf Fe concentrations under bic stress. According to previous investigations, this is due to the ability of this deme to mobilize Fe by the exudation of coumarins [23] acting as Fe reductants and chelators [31].

The analysis of fitness parameters and ionomic data revealed clear differences in the tolerance of *A. thaliana* plants to alkaline pH per se and to bic. In the sensitive accessions, Col-0 and T6_(c−)_, bic was more toxic than pH 8.3. T6_(c−)_ evolved on siliceous soil with extremely low carbonate content. Contrastingly, A1_(c+)_, which has evolved on a moderately carbonated soil (12.13% carbonate content) with pH 7.36, was more tolerant to bic than to pH 8.3. Soil conditions can act as driving force for local adaptation [23] and the carbonate/bicarbonate concentration in the original soil of A1_(c+)_ was strong enough to allow this accession to withstand the bicarbonate concentration provided in the nutrient solution (10 mM), yielding a pH of 8.3. However, this pH value achieved by organic buffer was harmful. It has previously been shown that buffers used to simulate alkaline stress may injure plants due to high salinity levels [32]. This was not the case here. The electric conductivity of both solutions was similar. We hypothesize that in the bicarbonate-tolerant deme, the buffer may not be as efficient as bic in triggering the expression of key genes required for nutrient acquisition and growth under bicarbonate stress.

### 2.3. Analysis of RNA-Seq Data

RNA-seq analyses were performed to characterize, at the gene expression level, the mechanisms underlying the differential response to bicarbonate of the two naturally selected accessions. Early responses were assessed by using leaf samples after 3 and 48 h exposure when the plants still did not exhibit any foliar symptoms of the stress treatments.

After Illumina sequencing, 72 library reads were obtained. Approximately 24,441 genes were detected to be expressed in each library (see Supplementary Datasets S2 and S3 for more details). To verify the RNA-seq transcriptome data, we selected eight genes with highly different expression levels in both demes after 3 h. The expression levels of the selected genes were quantified using q-PCR. Results obtained from both techniques were compared and we obtain high correlation between the expression level of the genes tested (Figure S2).

**Figure 2:**
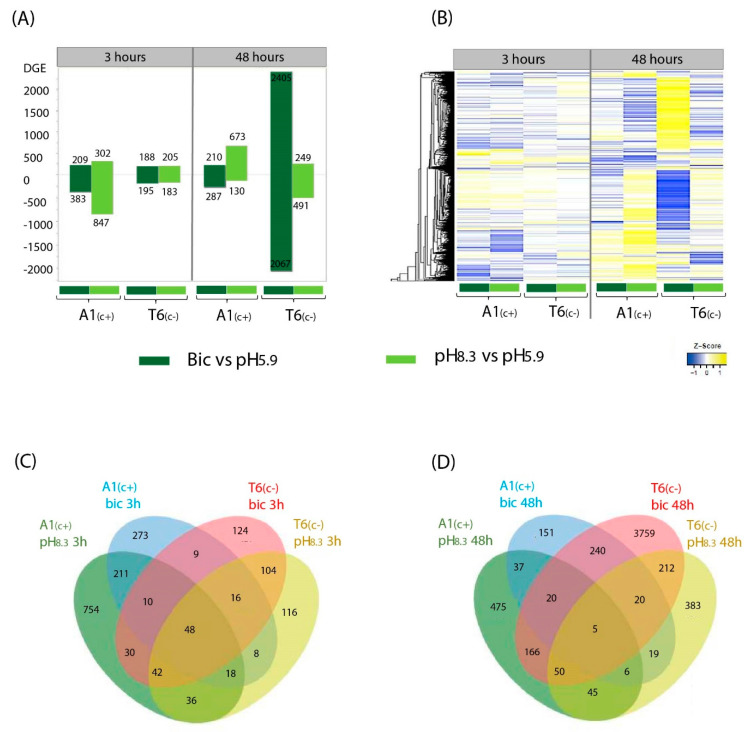
Compared transcriptomic profiles under different alkalinity treatments at two time points. Differential expressed genes (DEGs) bar plot (**A**), heatmap profile (**B**), and Venn diagram at 3 h (**C**) and 48 h (**D**) of the pairwise comparations pH 8.3 vs. pH 5.9 and bic vs. pH 5.9 in A1_(c+)_ and T6 _(c−)_ demes. DEGs were filtered at log fold change (LFC > 1, LFC < −1), and adjusted *p*-value < 0.05. Yellow areas indicate upregulated genes and blue areas indicate downregulated genes.

Annotated genes were filter by log fold change (LFC > 1 and LFC < −1) and adjusted *p*-value < 0.05. To understand the differences between A1_(c+)_-tolerant and T6_(c−)_-sensitive lines in response to different treatments (pH 8.3 vs. pH 5.9 and bic vs. control pH 5.9) at two time points (3 h and 48 h), we performed pairwise comparisons. After filtering, a total of 6163 differentially expressed genes (DEGs) were identified, considering accession, time, and treatment (Figure 2). In general, the bic treatment caused a higher number of DEGs (5205 genes) than the high pH treatment (2420 genes), indicating that bicarbonate stress involves more complex processes than simply the specific responses to alkaline pH (Figure 2A). Exposure for 3h to bic induced differential expression of a higher number of genes in A1_(c+)_ than in T6_(c−)_. Contrastingly, the highest number of DEGs was observed in the carbonate-sensitive accession T6_(c−)_ when exposed for 48 h to bic (Figure 2A). Gene expression profiles can also be compared in the heatmap plots shown in Figure 2B. Many of these DEGs that either were upregulated or downregulated by bic were not, or were only slightly, affected by pH 8.3.

### 2.4. Gene Ontology (GO) and Kioto Encyclopedia of Genes and Genomes (KEGG) Pathways under Bicarbonate Stress

The general overview of differential gene expression comparing bic versus pH 8.3 reveals that a greater number of genes and pathways were specifically implicated in the bic responses. Moreover, A1_(c+)_ and T6_(c−)_ showed contrasted reactions toward bicarbonate stress. For this reason, we further focused our analysis on the DEGs and pathways found in the plants exposed to the bic treatment.

Venn diagrams drawn for DEGs after 3 h (Figure 2C) and 48 h (Figure 2D) visualized DEGs that are shared between lines and time points under the different treatments. Within each deme, some of the DEGs were common to both bic and pH 8.3. This further supports the view that high pH alone cannot simulate bicarbonate stress. After 3 h exposure to bic, more genes were differentially expressed in A1_(c+)_ than in T6_(c−)_. Venn diagrams for DEGs comparing exposure times and demes for either pH 8.3 or bic are shown in Figure S3A,B, respectively. DEGs from each time point, treatment, and deme were mapped into the KEGG pathway and Gene Ontology (GO) terms to analyze their biological functions, molecular functions, and cell localization (Figure S3C,D, Datasets S4–S6).

The highest log fold discovery rate (FDR) and number of DEGs corresponded to 48 h bic exposure of T6_(c−)_. Most affected genes were involved in biological regulation, cellular and metabolic processes, response to stimulus, cellular components, and catalytic activity. After 3 h exposure, genes related to fatty acid elongation and cutin, suberin, and wax biosynthesis were distinctive in the sensitive deme. Contrastingly, in the tolerant A1_(c+)_, genes related to 2-oxocarboxylic acid, alpha-linolenic acid, flavonoid biosynthesis, and glyoxylate and dicarboxylate metabolism were differentially regulated after 3 h exposure to bic. After 48 h, in this deme, genes related to carotenoid biosynthesis, photosynthesis, nitrogen metabolism, and carbohydrate metabolism were mostly affected.

**Figure 3:**
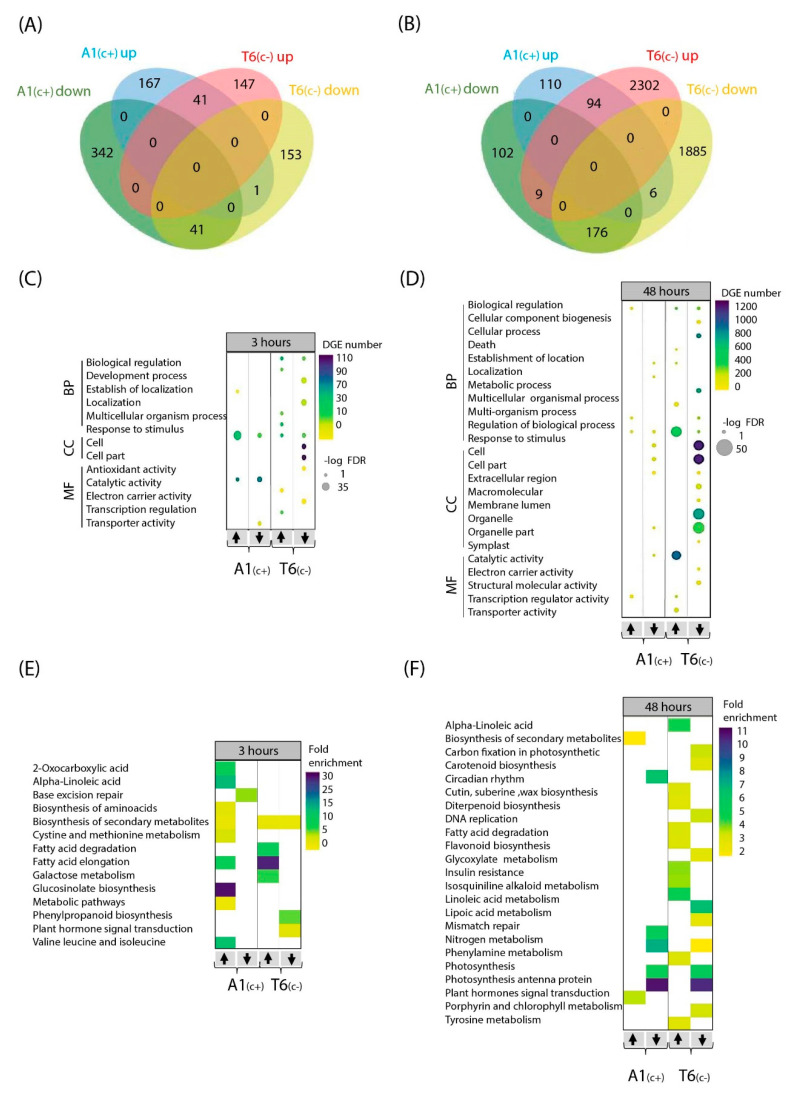
Gene Ontology (GO) and Kyoto Encyclopedia of Genes and Genomes (KEGG) pathway of bic vs. pH 5.9 treatments DEGs at two time points. Venn diagram of up- and downregulated DEGs in A1_(c+)_ and T6_(c−)_ comparing bic vs. pH 5.9 treatments after 3 h (**A**) and 48 h (**B**). Bubble plots indicating significant GO analysis of differentially expressed genes in bic vs. pH 5.9 comparison between A1_(c+)_ and T6_(c−)_ at 3 h (**C**) and 48 h (**D**). GO were filtered to adjusted *p*-value < 0.05. Scale colors indicate number of DEGs while bubble size indicates -log of adjusted *p*-value. GO terms were separated into biological function, cellular component, and molecular function. Arrows indicate up or downregulated genes. Heatmaps of KEGG pathway analysis from DEGs in bic 8.3 vs. pH 5.9 comparison between A1_(c+)_ and T6_(c−)_ at 3 h (**E**) and 48 h (**F**). KEGG pathway terms were filtered by *p*-value < 0.05. Scale colors indicate pathway fold enrichment. BP, biological process; CC, cellular component; MF, molecular function.

Venn diagrams comparing upregulated and downregulated DEGs between demes after 3 h bic exposure revealed that both demes only shared a small number of genes regulated in the same direction—41 up and 41 downregulated (Figure 3A). Differentially up- and downregulated genes in A1_(c+)_ were mainly related to response to stimulus and catalytic activity (Figure 3C). Interestingly, in T6_(c−)_, most affected genes were related to biological processes (up and down), cellular components (down), and electron carrier activity (down). The 3 h exposure to bic caused upregulation of many metabolism-related genes in both demes (Figure 3C). Most notably, in the tolerant A1_(c+)_, a 30-fold enrichment in the upregulation of genes involved in glucosinolate biosynthesis was observed. Enrichments between 5- to 15-fold in upregulated genes related to 2-oxocarboxylic acids; alpha linolenic acids; fatty acid elongation; and valine, leucine and isoleucine were found. Some enrichment was further seen in upregulated genes of amino acid biosynthesis, biosynthesis of secondary metabolites cysteine and methionine metabolism, and other metabolic pathways. Downregulation in A1_(c+)_ mostly affected genes related to base excision repair. In the sensitive T6_(c−)_, the most conspicuous enrichment concerned upregulation of genes related to fatty acid elongation, fatty acid degradation, and galactose metabolism (Figure 3C). Both upregulation and downregulation of genes related to secondary metabolism was observed. Downregulation also affected genes related to phenylpropanoid biosynthesis and plant hormone signal transduction (Datasets S7–S9).

The large amount of DEGs in T6_(c−)_ after 48 h exposure to bic (Figure 3B,D,F) indicated that this sensitive deme suffered from severe stress, leading to multiple secondary stress responses affecting almost all cellular and metabolic processes, even before visible foliar symptoms were detectable. Therefore, our further analytical efforts were focused on the differential responses between demes after 3 h exposure to bic (Datasets S7–S9).

### 2.5. Signal Perception and Signal Transduction

#### 2.5.1. Receptor-Like Kinases and Receptor-Like Proteins (RLK/RLP)

**Figure 4:**
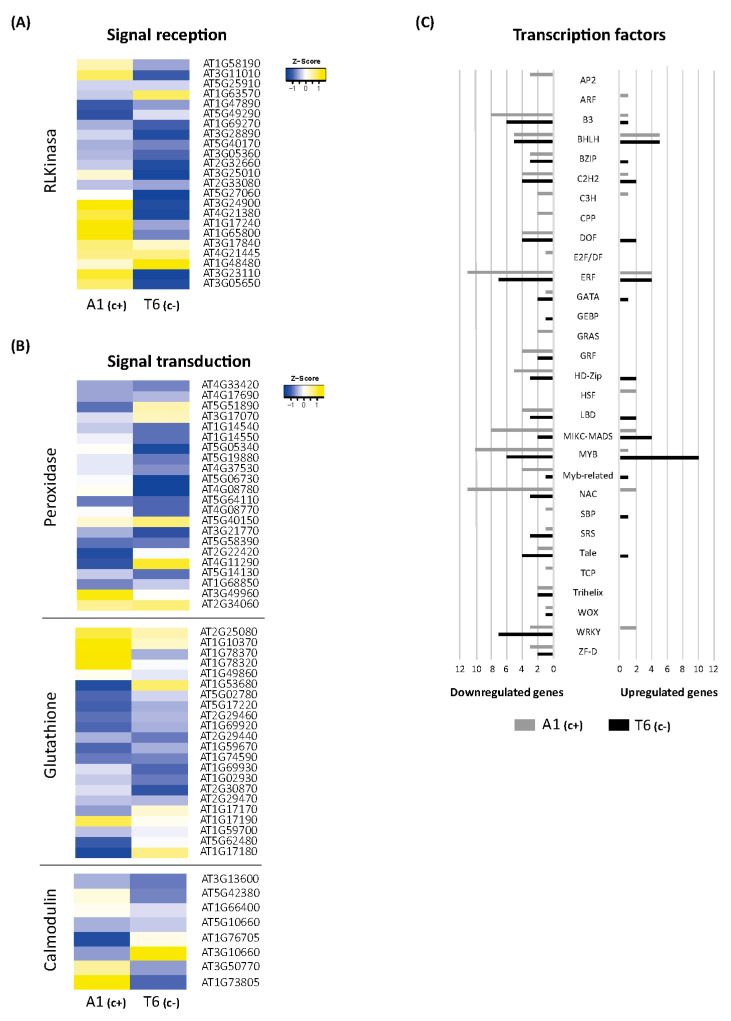
Differential expression of signal perception, transduction, and transcription factor (TF) genes involved in bicarbonate short responses. Heatmaps of DEGs from the receptor-like kinase gene family (**A**), and peroxidase, glutathione, and calmodulin gene families (**B**) in A1_(c+)_ and T6_(c+)_, comparing bic vs. pH 5.9 exposure for 3 h. Yellow color represents upregulated genes while blue color represents downregulated genes. (**C**) Bar plot indicating the total number of DEGs upregulated (right) or downregulated (left) within each transcription factor family. Tolerant deme A1_(c+)_ is marked in grey, while sensitive deme T6_(c−)_ is visualized in black color.

The 3 h exposure to bic caused a considerable up- and downregulation of DEGs related to response to stimuli in both A1_(c+)_ and T6_(c−)_. According to that, extreme DEGs among demes were selected (Dataset S10). Many of these genes are related to signal perception and signal transduction. Therefore, heat maps for these genes were drawn (Figure 4A,B).

Specific analyses of genes mediating Fe deficiency-induced reversible protein phosphorylation have revealed that in *A. thaliana* roots, the predominant gene groups code for proteins of the leucine-rich repeat kinase subfamily (RLK) [33]. Here, we found that 21 *RLK/RLP* genes were differentially expressed in shoots after 3 h exposure to bic (Figure 4A). In A1_(c+)_, bic distinctively upregulated genes coding for receptor-like proteins RLP 2, 32, 34, 37, and 39, and for receptor-like serine/threonine protein kinases SD1-6, also designed as ARK2, and SD18 (Figure 4A). Among these different RLPs, only RLP32 belongs to the validated defense RLPs [34]. This protein recognizes Inf-1, the bacterial translocation initiation factor of proteobacteria. Both RLP32 and RLP39 have been proposed to interact with SOBIR1; the interaction with SOBIR1 may be mediated via a stretch of negatively charged amino acids, such as aspartate and glutamate, in the apoplast close to the plasma membrane. Contrastingly, *RLP2*, also known as Clavata2-related gene, and the serine/threonine kinases SD1-6 or ARK2 are involved in developmental processes [35].

In both demes, bic upregulated *RKL1, RLK902*, and *CRR9*. RKL1 and RKL902 are two highly homologous receptor kinases apparently involved in mechanical and biotic stress signaling and salicylic acid-induced responses [36]. CRR9, chlororespiratory reduction 9, is a protein required for the formation of a NADH dehydrogenase-like complex [37]. The protein is involved in cyclic electron transport around photosystem I, a process favored under Fe deficiency [38]. In T6_(c−)_, bic specifically upregulated CRRSP7, a putative cysteine-rich repeat secreted protein. All other *RLK/RLP* genes were downregulated by bic in both demes.

#### 2.5.2. Peroxidases

Most of the 22 genes related to peroxidases, which were differentially expressed after 3 h bic treatment, were downregulated in both demes (Figure 4B). This is in line with reduced activity of cell wall peroxidase observed by others in Fe-deficient plants [39]. Downregulated only in T6_(c−)_ were *PER4, 5, 38, 54,* and *58*, coding for extracellular peroxidases involved in oxidative stress; PER52 involved in lignin biosynthesis; and PER37 located in vascular bundles acting as a negative regulator of growth [40]. However, in the sensitive T6_(c−)_, *PER39*, *63*, and *66* were specifically upregulated. The corresponding peroxidases are also located in the apoplast and are related to oxidative stress. PER66 has been found to be involved in xylem formation and lignification [41]. Specifically downregulated in A1_(c+)_ were *PER11*, *39*, and *66*, which are related to detoxification of reactive oxygen species (ROS), and *PER17*, involved in lignin biosynthesis. Interestingly, *PER35* was specifically upregulated in A1_(c+)_. PER35 has been associated with transcription-associated mark trimethylation of H3 lysine 4 (H3K4me3) [42] and is essential for gene regulation during cell transition processes [43]. Taken together, these results reinforce the crucial role of differential expression of peroxidase genes in the early leaf responses to bicarbonate. Recent research by Santos et al. (2019) [44] hypothesized that Fe-efficient plants do not activate the antioxidant machinery in leaves because of better Fe translocation to the shoots, while inefficient plants suffering from oxidative stress induced by Fe deficiency show reduced ascorbate peroxidase (APX) activity.

#### 2.5.3. Glutathione-Related

In both A1_(c+)_ and T6_(c−)_, exposure to bic upregulated GPX1 (Figure 4A). This chloroplastic glutathione peroxidase plays an important role in degradation of H_2_O_2_ under stress conditions [45]. Under bic exposure, main DEGs related to glutathione were glutathione-S-transferases (GSTs) of the tau type (GSTU). Most of these *GSTU* genes were downregulated in both demes (*GSTU3*, *4*, *6*, *10*, *11*, *12*, and *15*) (Figure 4A). Specifically upregulated in A1_(c+)_ were *GSTU17* and *GSTU26*, while *GSTU25* and *GSTU28* were specifically upregulated in T6_(c−)_. GSTs of the phi type, namely, *GSTF6*, *10* and *12*, were downregulated in both demes. The functions of GSTUs are poorly explored. They are generally described as being associated with toxin catabolic processes. Different *GSTUs* (*GSTU1*, *3*, *4*, *8*, *10*, *11*, *12*, and *24*) have been found strongly upregulated after fungal or bacterial attack [46]. Interestingly, certain GSTUs and GSTFs have been involved in the glucosinolate metabolism of Brassicaceae [47,48,49]. GSTF6 and GSTU4 seem to catalyze the GSH conjugation of indole-3-acetonitrile, a step in camalexin biosynthesis, while GSTF10 has been proposed to act in the biosynthesis of indolyl-glucosinolates. GSTU20 may act as a GSH donor in the biosynthesis of aliphatic glucosinolates [50].

Bic specifically upregulated *GSTU25* and *28* in T6_(c−)_ (Figure 4A). GSTU strongly binds to hydroxy C6 conjugates of fatty acids and it has been proposed that this enzyme may catalyze the formation of GSH conjugates of fatty acids and promote the transport to other compartments [51]. The differential expression of genes coding for glutathione-S-transferases under bic exposure suggests a general downregulation of glucosinolate biosynthesis in T6_(c−)_ and a shift from indolyl-GS to aliphatic GS in the tolerant A1_(c+)_ deme.

#### 2.5.4. Calmodulin

Bicarbonate exposure negatively regulated the IQ motif-containing protein IQM2 and the calmodulin-like protein coded by *At5g10660* in both demes (Figure 4A). Specifically downregulated in T6_(c−)_ were *CML23*, *CML37*, *CML41*, and *SARD1* (SAR deficient 1). Instead, in A1_(c+)_, both *SARD1* and *CML41* (calmodulin-like 41) were specifically upregulated. The calcium-dependent protein kinase 2 (CPK2) was specifically upregulated only in T6_(c−)_ and downregulated in A1_(c+)_. These results indicate clear differences in early Ca^2+^-dependent signaling pathways between the bic-tolerant and the sensitive deme. Specific upregulation of *CPK2* in T6_(c−)_ suggests enhanced phosphorylation of nicotinamide adenine dinucleotide phosphate (NADPH) oxidases inducing ROS signaling and programmed cell death [52]. However, in this bic-sensitive deme, both jasmonate (JA) and salicylate (SA) signaling pathways seemed downregulated. This is supported by the observed downregulation of *CML37,* required for jasmonic acid (JA) production [53], and of *SARD1*, coding for a key transcription factor in salicylic acid (SA) biosynthesis [54] Contrastingly, in A1_(c+)_, bic-induced enhancement of both *SARD1* and *CML41* indicates activation of biotic stress signaling. CML41 can be induced by flagellin and promotes the deposition of callose in plasmodesmata, thus conferring resistance to *Pseudomonas syringae* [55]. Interestingly, studies with SA-deficient mutants have shown that accumulation of endogenous SA levels are required for activation of the Fe deficiency response. However, under Fe deficiency, *pad4*, a mutant with deficient SA biosynthesis, maintained higher shoot Fe levels and higher chlorophyll concentrations than Col-0 wildtype [48].

#### 2.5.5. Transcription Factors

Exposure to bic had a strong influence on DEGs coding for transcription factors (TFs) (Figure 4C). Surprisingly, most were downregulated. In A1_(c+)_, 22 TFs were upregulated and 105 were downregulated; in T6_(c−)_, upregulation and downregulation were 37 and 70, respectively. Among the TFs exclusively upregulated in the tolerant A1_(c+)_, we found the *Auxin Response Factor*, coding for ARF20, which acts as repressor of gene transcription in response to auxin in the shoot apical meristem [56]. Upregulated only in A1_(c+)_ were also two *Heat Shock Factors—At4g19630* coding for an unknown protein, and *At2g26150* coding for HSFA2, which responds not only to heat stress, but also to salinity and osmotic imbalance [57]. Among C3H type TFs, one coding for ZDW4 with unknown function was upregulated, while those coding for C3H14 and CDM1 were downregulated. Interestingly, C3H14 and CDM1 are regulators of the biosynthesis of hemicellulose and callose, respectively [58,59].

Specifically downregulated by bic in A1_(c+)_, but unaffected in T6_(c−)_, were the following TFs: three *Apetala2* (*AP2s*), coding for AIL7, ANT, and WRK14; two *Cysteine-rich polycomb-like (CPPs)*, coding for TCX2 and TCX6; one *E2F/DF*, coding for DEL3; and one *Teosinte branched1/Cincinnata/proliferating cell factor TCP*, coding for TCP1 (Figure 4C). Except for WRK14, which is involved in the control of cuticular wax biosynthesis under stress, all others are related to cell proliferation and shoot growth regulation. AIL7 is required for high PIN1 (auxin efflux carrier component1) responsible for leaf shape, ANT controls cell proliferation, TCX2 has been involved in fate transition and cell division in stomatal development, and DEL3 plays a role in cell elongation and differentiation [60]. This specific downregulation of TFs involved in plant growth promotion indicated that under bic-exposure, the shoot growth is quickly adjusted to the stress situation. However, this downregulation after 3 h did not result in reduced shoot growth after longer term exposure. Most probably, the downregulation is a sign of transitory changes leading to an adaptive growth response, allowing better performance under bic than in the sensitive T6_(c−)._

In both demes, five TFs of the basic *helix-loop-helix family* (*bHLH)* were upregulated and five were downregulated. Several TFs of this family play a central role in Fe homeostasis under Fe deficiency conditions [61,62,63]. However, under bic exposure only *PYE* was upregulated in both demes. PYE is a key regulator of plant Fe deficiency responses. *PYE* is a strong repressor of *FER* coding for ferritin, which tightly binds Fe. *FER* repression thus reduces Fe sequestration and enhances the Fe fraction available for essential cell processes [62]. Contrastingly, only in T6_(c−)_ were bHLHs of the Ib clade, namely, *bHLH39*, *38*, *100*, and *101*, were upregulated by bic. These TFs activate FIT, the key regulator for Fe uptake and transport. Furthermore, Ib clade bHLHs promote leaf expansion in the shoot [63]. FIT repressor bHLH11 was downregulated in T6_(c−)_.

In A1_(c+)_, upregulated bHLHs were *bHLH23*, *114*, and *136 (PRE1),* while *bHLH10* and *45* (MUTE), *SPCH*, and *HEC1* were downregulated. These TFs seemed to not be directly related to Fe deficiency response pathways but to growth and developmental processes. *PRE1* mediates brassinoesteroid regulation of cell elongation [64]. HEC1 targets WUS and promotes cell proliferation in the shoot apical meristem [65]. SPCH and MUTE are key regulators for stomatal size and density in response to environmental changes [66].

A highly contrasting influence of bic on MYB TFs in both demes was observed. Only *HOS10*, a R2R3-type MYB TF, was upregulated in A1_(c+)_. Nine other MYBs were downregulated (*MYB1*, *10*, *23*, *46*, *53*, *86*, *120*, and *122*, and *MYB3-R4*). *MYB3-R4* is a transcriptional activator of cytokinesis [67]. MYB122 is required for indolyl-glucosinolates acting downstream of JA, SA, ethylene, and abscisic acid (ABA) [68]. These results further confirm that A1_(c+)_ exposure to bic has a quick influence not only on shoot growth and developmental processes, but also on biotic stress signaling pathways.

A1_(c+)_ exposure to bic enhanced the transcription of *WRKY70*, *54*, and *18*, while *WRK23*, *44* (TTG2), and 45 were downregulated. Inversely, in T6_(c−)_, only downregulation of WRKYs was observed, namely, *WRKY12*, *14*, *30*, *50*, *51*, *62*, and *71*. WRKYs participate in regulatory networks of biotic and abiotic stress responses, either activating or repressing transcription [69]. In biotic stress signaling, SA-induced WRKYs can repress JA signaling, either directly or indirectly [70]. Overexpression of SA-induced *WRKY70* represses the expression of *PDF1.2* induced by MeJa [71,72]. *WRKY23* regulates auxin transport by a local regulation of flavonol biosynthesis [73].

Among the *Ethylene Responsive Factors (ERFs)*, 4 were upregulated and 11 were downregulated in A1_(c+)_ (Figure 4C). In T6_(c−)_, upregulation and downregulation were 4 and 7, respectively. Among the upregulated ERFs in A1_(c+)_, we found *ERF13*, *98*, and *109*. *ERF13* responds to exogenous ABA and JA and increases expression of the defensin gene *PDF1.2* [74]. ERF98 has been related to ascorbic acid biosynthesis [75], while ERF109 acts downstream of auxin, promoting cell growth under stress conditions [76]. Further influence of bic on growth regulation in A1_(c+)_ is indicated by the downregulation of *Cytokinin Response Factors* (*CRF*)*1, 2, 5,* and *6.* Disruption of these TFs yields larger leaf rosettes and delay of senescence [77]. Interestingly, *ERF72* was also downregulated in the tolerant deme. *ERF72* has been described as quickly upregulated under Fe deficiency; the enhanced expression has been related to the regulation of chlorophyll degradation and Fe deficiency-induced chlorosis [78]. TFs related to drought resistance were also downregulated in A1_(c+)_, such as *Translucent Green (TG),* an activator of aquaporins [79]; *DREB19* [80]; and *SHINE2*, involved in epicuticular wax production. Differently, in T6_(c−)_, bic induced upregulation of stress-related ERFs—*ERF54* and *ESE2*—and *ERF39* related to primary cell wall biosynthesis [81]. Contrastingly, downregulation of *ESR1* (*Enhancer of Shoot Regeneration 1*); stress-related *ERF14*; and *ERF96*, a positive regulator of ABA response [82], was observed.

Bic treatment caused downregulation of eleven NAC TFs in A1_(c+)_; only *NAC44* and *NAC95* were upregulated. In T6_(c−)_, only *NAC71*, *90*, and *95* were downregulated. Most NACs are involved in plant developmental processes, phytohormone signaling, and biotic and abiotic stress responses [83]. *NAC90* is one of the “NAC troika genes” that act as regulators of the shift to leaf senescence. *NAC42* and *87* are targets of this NAC troika [84].

The considerable differences between A1_(c+)_ and T6_(c−)_ in the bic-induced changes in TF expression reveal quick regulation of growth and developmental processes in the tolerant deme in response to bic. These adaptive responses are mainly orchestrated by SA, JA, auxin, and ABA and induced by biotic and abiotic stress signaling pathways. Upregulation of PYE helping to mobilize Fe from ferritin was the only typical Fe deficiency response in A1_(c+)_. Contrastingly, in the sensitive T6_(c−)_, bic exposure caused fast FIT-regulated Fe deficiency responses in line with the impaired ability to translocate Fe to the developing shoots.

### 2.6. Protein–Protein Interaction Network Functional Enrichment Analysis with Specific Bic-Induced Genes

**Figure 5:**
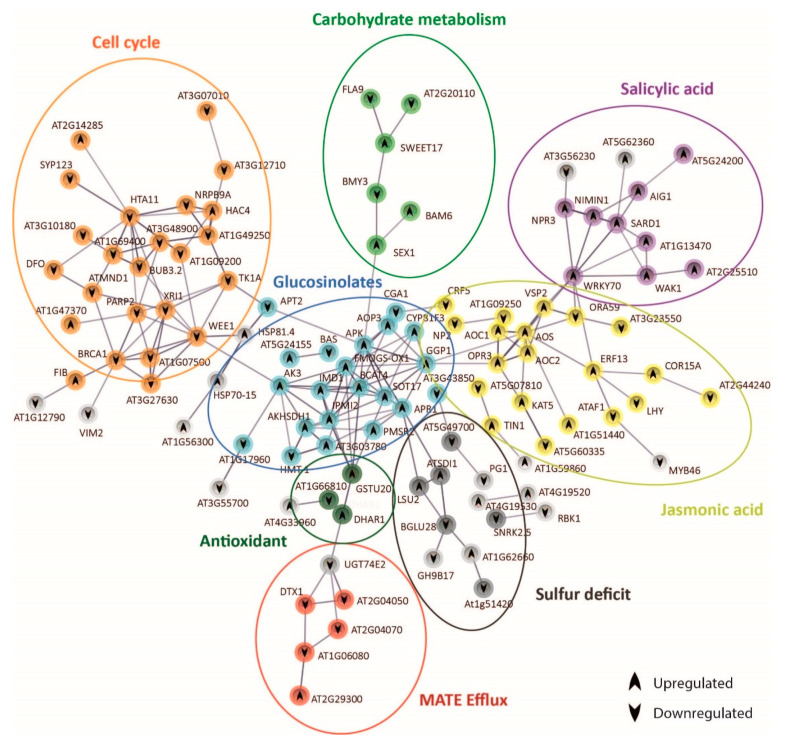
Protein–protein interaction network functional enrichment analysis of specific genes derived from the response to bicarbonate of A1 _(c+)_. Gene protein interaction network of A1_(c+)_ exclusive DEGs from bic vs. pH 5.9 comparison after 3 h exposure. Each sphere corresponds to one gene and nodes represent protein interactions. Gene pathways are shown in different colors. Arrows indicate up- or downregulation of the genes.

**Figure 6:**
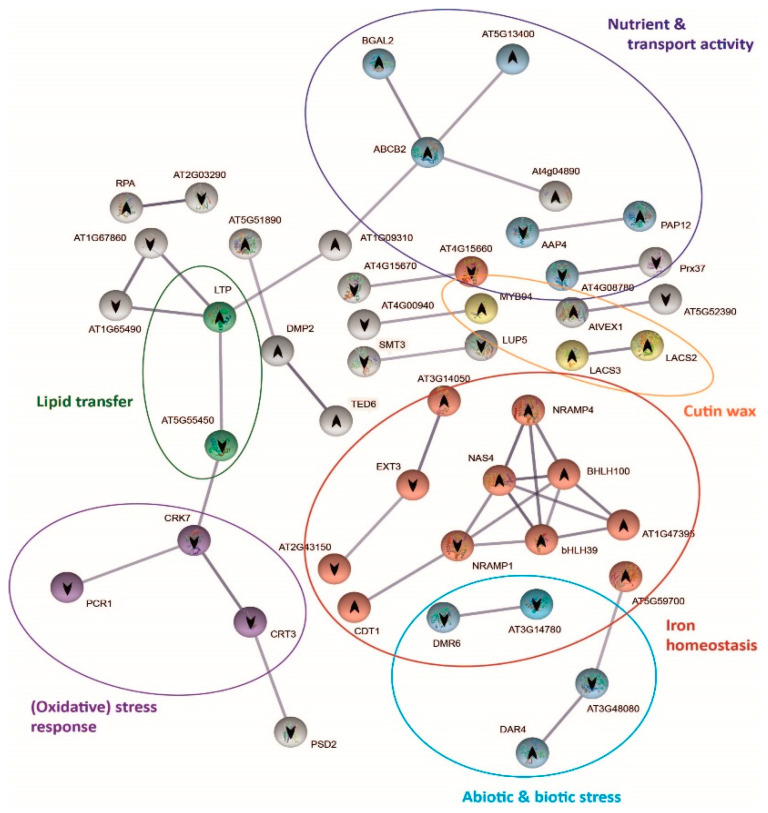
Protein–protein interaction network functional enrichment analysis of specific genes derived from the response to bicarbonate of T6 _(c−)_. Gene protein interaction network of T6_(c−)_ exclusive DEGs from bic vs. pH 5.9 comparison after 3 h exposure. Each sphere corresponds to one gene and nodes represent protein interactions. Gene pathways are shown in different colors. Arrows indicate up or downregulation of the genes.

The analysis of the expression of genes coding for stress signal perception/transduction and transcription factors revealed that shoots of both demes perceived and responded to carbonate stress in different ways. To further explore these differential mechanisms, we performed protein–protein interaction network functional enrichment analysis using STRING database (Figure 5 and Figure 6). After 3 h exposure to bic, 273 genes were differentially expressed in A1_(c+)_ and 123 genes in T6_(c−)_. In both demes, part of the genes was not linked to others due to lack of information on these protein interactions (Datasets S11 and S13). Nonetheless, we could observe clear differences between the tolerant A1_(c+)_ (Figure 5) and the sensitive T6_(c−)_ (Figure 6). In A1_(c+)_, many of the DEGs could be grouped into seven interconnected, functional categories related to SA, JA, GS, cell cycle, carbohydrate metabolism, sulfur deficit, antioxidants, and multidrug and toxic compound extrusion (MATE) efflux (Figure 5, Dataset S12). Inversely, in the sensitive T6_(c−)_, the main DEGs were grouped into five categories: Fe homeostasis (up); abiotic and biotic stress response; cuticular wax, nutrient, and transport activity; lipid transfer; and oxidative stress response. Only nutrient transport activity, lipid transfer, and oxidative stress response were connected by STRING (Figure 6, Dataset S14).

Shen et al. (2016) [48] reported that Fe deficiency induces SA accumulation, which enhances auxin and ethylene signaling and activates Fe transport by *bHLH38/39* regulation of downstream iron genes. In fact, in the sensitive deme T6_(c−)_, both the *FIT*-dependent [85] and the *FIT*-independent pathways [86] for iron homeostasis were activated by bic, as indicated by the upregulation of *bHLH39* and *bHLH100*, respectively. Upregulation of *NAS4* coding for nicotianamine synthase and *NRAMP4* coding for Fe transporter exporting Fe from vacuoles further confirms the quick activation of Fe mobilizing mechanisms in the bic-sensitive deme.

In the tolerant A1_(c+)_, bic enhanced SA, as shown by the upregulation of *SARD1* [54] and several SA-induced genes such as *WAK1* and *At2g25510*. SA, in turn, induced *WRKY70*, a key regulator of SA-induced genes and a repressor of JA-responsive genes [70]. In our tolerant plants, bic exposure caused a downregulation of *ORA59*, which is essential for the integration of JA/ethylene transduction pathways. *ORA59* is required for the expression of defensin *PDF 1.2*, a typical JA signaling gene [87]. Contrastingly, *VSP2*, another JA-responsive marker gene, as well as *AOS*, *AOC1,* and *OPR3* involved in JA biosynthesis, were upregulated (Figure 5). STRING connected AOS and OPR3 to GGP1, a γ-glutamyl peptidase that catalyzes the hydrolysis of the γ-glutamyl residue of GSH-conjugated glucosinolates. GSH is the sulfur donor for GS biosynthesis and GGP1 is a key enzyme in this process [88]. Upregulation of *BCAT4*, *FMOGS-OX1*, *AKHSDH1*, *IMD1*, and *GSTU20* is in line with a bic-induced enhancement of aliphatic GS. There is a close connection between mineral nutrient supply and GS biosynthesis [89]. Sulfur deficit specifically reduces the production of aliphatic GS. Here, we observed an upregulation of *SDI1* (*Sulfur Deficit Induced 1*) and *LSU2* (*Response to Low Sulfur*). *SDI1* has been reported to promote the release of sulfur from internal storage sites [90]. *LSU2* is upregulated not only under S-deficiency, but also by Fe deficiency, high pH, and Cu toxicity [91]. Interestingly, *BGLU28* was downregulated. This myrosinase gene usually is upregulated under sulfur deficiency, while *bglu28* mutants display high GS levels [92]. Accordingly, our data indicate that in the tolerant deme A1_(c+)_ bic does not directly cause S deficiency but specifically induces GS production and, consequently, the upregulation of S-deficiency related genes to cope with the higher S-demand.

STRING connected GS genes to cell cycle and DNA-repair related genes by *AK3* and *WEE1* (Figure 5). *AK3* codes for an aspartate kinase catalyzing the first step in aspartate-derived amino acids [93]. WEE1 is a kinase involved in cell cycle inhibition related to DNA damage and repair [94]. Surprisingly, exposure to bic downregulated many genes involved in DNA repair, such as *XR1*, *BUB32*, *BRAC1*, and *MND1*. DNA damage repair is usually upregulated in plants exposed to different abiotic and biotic stresses [95]. Downregulation of *WEE1* together with enhancement of *FIB* coding for tryptophan aminotransferase, a key enzyme in auxin biosynthesis [96], supports the view that leaf cell growth was activated after 3 h exposure to bic. These results further indicate that in the tolerant A1_(c+)_ adaptive shoot growth events are activated in response to bic. However, RNA-seq only provides a snapshot of gene expression at a given time point; here, 3 h. It is possible that this growth promotion is a consequence of an earlier cell cycle arrest and upregulation of DNA repair in response to stress, followed by a downregulation after successful correction. To prove this attractive hypothesis, one must perform expression analysis after shorter exposure time. In addition, STRING connected GS biosynthesis to carbohydrate metabolism via adenosine 5′-phosphosulfate kinase (APK) and *SEX1*, a α-glucan water dikinase required for starch degradation. Enhanced starch degradation is also supported by the upregulation of *BAM6* coding for ß-amylase, while *BMY3* coding for α-amylase was downregulated. Glucose has been identified as a positive regulator of GS biosynthesis [97]. Furthermore, GS biosynthesis was related to organic carbon from 2-oxocarboxylic acid metabolism via *AK3*.

DTX/MATE efflux carrier expression was downregulated by bic exposure. STRING connected downregulated *DTX1* and *DTX3* (*At2g04050*) to upregulated *DHAR1* (deshydroxyascorbate reductase) by means of the glucosyltransferase gene *UGT74E2*. Ascorbic acid not only plays a key role in the protection against oxidative stress, but also improves internal Fe availability [98]. The functions of DTX efflux carriers are still poorly established. DTX1 has been involved in cadmium tolerance [99]. DTX3 may facilitate membrane transport of xenobiotics; both DTX3 and DTX4 (*At2g04070*) seem to have an efflux protein with broad substrate specificity [100]. Downregulation of these DTXs could be related to the reduced efflux of some organic compound acting as a strong ligand for Fe, thus facilitating Fe availability.

### 2.7. Mechanisms Underlying Differential bic Toxicity and Tolerance in A. thaliana Demes

Our results demonstrate that the effects of bicarbonate on *A. thaliana* differs from those caused by high pH alone (Figure 1). At equal pH, bic is more toxic to sensitive T6_(c−)_ and Col-0 than an alkaline solution with organic buffer. In the tolerant A1_(c+)_, the opposite was found. Induction of Fe deficiency is among the best-known consequences of bicarbonate toxicity in sensitive plants. In fact, the quick upregulation of Fe homeostasis-related genes in leaves of T6_(c−)_ exposed to bic (Figure 6) is a clear sign of the difficulty of these sensitive plants to maintain sufficient Fe transport to the leaves. Bicarbonate not only interferes with the Fe-reducing capacity of the roots [101], but also specifically inhibits the translocation from roots to shoots [102]. This inhibition of Fe translocation has classically been related to enhanced root production of organic acids, especially citrate. Citrate is a strong ligand for Fe and may favor Fe sequestration into root vacuoles [103]. More recently, studies with bic-exposed kiwi plantlets supplemented with ^57^Fe related bic-induced inhibition of Fe translocation to the leaves with apoplastic accumulation of water-soluble Fe in the cell wall apoplast and the imbalance of nitrogen and carbon metabolism [98]. In fact, due to carbonic anhydrase activity, HCO3^−^ supply favors dark fixation of inorganic C [104], leading to enhanced organic acid biosynthesis. In T6_(c−)_, bic exposure led to DEGs involved in nutrient balance and transport activities that were not directly related to Fe deficiency DEGs (Figure 6). Upregulation of *BGAL2*, coding for β-galactosidase, and downregulation of *EXT3* and *EXT21* (*AT2G43150*) suggests fast bic influence on leaf cell walls. Upregulation of *AT5G13400* coding for the nitrate transporter NRT1, as well as downregulation of *AAP4*, responsible for phloem export of amino acids, is in line with enhanced nitrogen requirement in bic-stressed leaves.

In the bicarbonate-tolerant A1_(c+)_, we have previously shown enhanced root exudation of flavonol-type phenolics enabling roots to mobilize sparingly soluble Fe [23]. Here, we further observed bic-induced upregulation of *PYE* and *Ascorbate Reductase* in leaves, both being factors that can contribute to increased availability of biologically active Fe in the leaves. To avoid imbalance of organic carbon caused by dark fixation of inorganic carbon derived from bic, A1_(c+)_ may shuttle organic C into the pathway of aliphatic glucosinolates, as indicated by the upregulation of aspartate kinase 3 *(AK3)* and 3-isopropylmalate dehydrogenase *(IMD1)* (Figure 6). Sulfur requirement for GS biosynthesis is provided by the upregulation of sulfur deficiency-induced genes such as *LSU2* and *SDI1* and the downregulation of *BGLU28* involved in GS degradation under S deficiency conditions.

A key question concerns the trigger mechanism and the regulation of these quick differential leaf responses in A1_(c+)_. According to our RNA-seq data, SA and JA may play a central role. Bic-induced upregulation of *WRKY70* connected the enhanced SA pathway to differential regulation of the JA pathway and GS biosynthesis (Figure 5). Interestingly, in the tolerant A1_(c+)_, bic exposure specifically enhanced the expression of certain defense-related RLKs and RLPs (Figure 4). A primary objective of future research is to explore whether apoplastic bic, less Fe translocation from root to shoot, or any other root-derived signal is responsible for the activation of these primary signal perception molecules that trigger defense mechanisms in the leaves of bic-tolerant A1_(c+)_.

## 3. Materials and Methods

### 3.1. Plant Material

In previous studies, natural variation of *Arabidopsis thaliana* populations from Catalonia [105] were tested in a multi-year small scale common garden under carbonated condition [23]. Seeds of extreme phenotype lines A1_(c+)_, a moderately alkaline-tolerant deme, and T6_(c−)_, an alkaline-sensitive deme, were collected from the last reciprocal transplant experiment performed in 2015 and stored under fresh (4 °C) and dry conditions until the beginning of the experiments. Col-0 seeds purchased from The Nottingham Arabidopsis Stock Centre (NASC, Nottingham, U.K.) [106] were included as a reference genome.

### 3.2. Growth Conditions

Seed sterilization: Selected seeds were surface sterilized by soaking in 70% (*v*/*v*) ethanol for 1 min, suspended in 30% (*v*/*v*) commercial Clorox bleach and 1 drop of Tween-20 for 5 min, and rinsed 5 times in sterile 18 MΩmilli-Q water.

Hydroponic culture: Seeds were sown in 0.2 mL tubes containing 0.6% agar prepared in nutrient solution 1/2 Hoagland (pH 5.9). Seeds were kept at 4 °C for 7 days in the dark to synchronize germination. Tubes containing seed were placed in the growth chamber (12 h light/12 h dark, 150 µmol cm^−2^·s^−1^, 40% humidity and 25 °C). After root emergence, the bottom of the tubes containing seedlings was cut off and the tubes were placed in 150 mL hydroponic containers with aerated nutrient solution 1/2 Hoagland (pH 5.9).

When 15 days old, the seedlings were separated in different sets and the following treatments were applied: control (½ Hoagland solution at pH 5.9), high pH (½ Hoagland solution at pH 8.3), and bicarbonate (½ Hoagland solution at pH 8.3 with 10 mM NaHCO_3_). Solutions were buffered with different proportions of MES (2-(N-morpholino) ethanesulfonic acid hydrate, 4-morpholineethanesulfonic acid) and BTP, depending on final pH in continuous aeration. The selected bicarbonate concentration has previously been shown to discriminate between the carbonate-tolerant and -sensitive deme [23]. To check whether this concentration allows reproductive growth in the tolerant accession, we performed A1_(c+)_ preliminary essays with bicarbonate concentrations ranging from 0 to 20 mM (pH 8.3). Rosette diameter and length of the longest root were monitored every week by image analysis (Figure S1).

Plate culture: For germination assays, 45 seeds from each deme were sown in plates under a flow cabinet with sterile material. Plates contained 3 treatments: control (½ MS at pH 5.9), high pH (½ MS at pH 8.3), and bicarbonate (½ MS at pH 8.3 and 10 mM NaHCO_3_). All plates contained Phyto-agar 0.6% (Duchefa, Haarlem, The Netherlands), and solutions were buffered using different proportions of MES and BTP depending on final pH. Plates with seeds were kept at 4 °C for synchronizing germination. After 7 days under stratification treatment, plates were moved to a growth chamber (12 h light/12 h dark, 150 µmol cm^−2^·s^−1^, 40% humidity and 25 °C). Germination and radicle emergence were daily checked during the following 10 days.

### 3.3. Physiological Trait Measurements

Plants from tolerant A1_(c+)_, sensitive T6_(c−)_, and Col-0 were used to test fitness in hydroponic conditions. Every week, pictures of the entire plants were taken. The photographs were used to measure the length of the largest root and the rosette diameter (*n* = 9–12) using ImageJ software [107]. We did not use a dye prior to image acquisition.

Siliques production was counted at maturity (*n* = 9). The leaf nutritional state of plants was assessed in plants submitted for 15 days to treatment conditions. Leaves were washed with 18 MΩ water before placing into Pyrex digestion tubes. Sampled plant material was dried for 4 days at 60 °C and weighed before open-air digestion in Pyrex tubes using 0.7 mL concentrated HNO_3_ at 110 °C for 5 h in a hot-block digestion system (SC154-54-Well Hot Block, Environmental Express, Charleston, SC, USA). Four plant replicates from each deme and treatment were used to determine the concentrations of the selected elements (Ca, K, Mg, Na, P, S, B, Mo, Cu, Fe, Mn, Zn) by inductively coupled plasma optical emission spectroscopy (ICP-OES; Thermo Jarrell-Ash, model 61E Polyscan, Waltham, MA, USA).

### 3.4. RNA Isolation, Library Construction, and Sequencing

Leaf material from plants cultivated under hydroponic conditions were recollected 3 and 48 h after starting the treatments. Twelve plants per line and treatment were pooled to perform 3 biological replicates. Leaves were immersed in liquid nitrogen, homogenized to a fine powder, and stored at –80 °C. The total RNA of 100 mg of leaf powder for each biological replicate was extracted using the Maxwell plant RNA kit (Promega Corporation, Madison, WI, USA) following the manufacturer’s instructions. For each sample, approximately 2 μg of total RNA was used for quality evaluation. Total RNA was quantified using a Qubit 2.0 Q32866 (Life Technologies, Carlsbad, CA, USA) to prepare the complementary deoxyribonucleic acid (cDNA) library.

cDNA library was composed by 72 samples, collected from A1(_c+_) and T6(_c−_) plants exposed for 3 h (quick response) or 48 h (long response) to the different treatments (2 biological replicates and 3 technical replicates). Library preparation was performed using Novogene protocol. The cDNA library was sequenced at the Illumina NovaSeq 6000 using standard procedures to generate paired end reads of 150 bp and number of analyzed read 30-47 million pairs (Novogene, Sacramento, CA, USA).

Clean reads were obtained by removing reads containing adapters, reads containing poly-N, and low-quality reads from raw data. Simultaneously, Q20, Q30, and GC content of the clean data were calculated. After filtering, clean reads on average had 35.7 million reads, which occupied 98% of the total reads of all libraries. In all samples, the Q30 value was over 89%, and the GC content was 44% (Datasets S2 and S3). All downstream analyses were based on the clean data with high-quality reads. Reference genome and gene model annotation files were downloaded from the genome website directly. Index of the reference genome was built using Bowtie v2.2.3 (John Hopkins University, Baltimore, MD, USA) and paired-end clean reads were aligned to the reference genome using TopHat v2.0.12 (John Hopkins University, Baltimore, MD, USA).

Differential expression analysis of samples was performed using a model based on the negative binomial distribution [108] and the DESeq R package (1.18.0) (www.Bioconductor.org). The resulting *p*-values were adjusted using the Benjamini and Hochberg’s approach [109] for controlling the false discovery rate (FDR). To perform the final list of DEGs, we filtered genes by adjusted *p*-value < 0.05 and LFC > 1 and LFC < −1. Raw reads and normalized gene expression of RNA-seq analysis were deposited in the Gene Expression Omnibus (GEO) database and in the National Center for Biotechnology Information’s (NCBI) Sequence Read Archive (GSE164502).

### 3.5. Gene Ontology, KEGG Pathway, and Functional Protein Association Network Analysis of DEGs

Gene Ontology (GO) enrichment analysis of DEGs was implemented by AgriGO V2 (GO Analysis Toolkit and Database for Agricultural Community (cau.edu.cn) [110]. Significant GO terms (*p* < 0.05) were classified into 3 categories: biological function, molecular process, and cellular component. KEGG pathway and STRING version 11.0 were used to understand high-level function and gene interaction network of differential expressed genes [111].

### 3.6. Relative Expression Analysis

To produce cDNAs from RNAs, we used the iScriptTM cDNA Synthesis Kit (Bio-Rad, USA) with 1 μL iScript Reverse Transcriptase + 4 μL 5x iScript Reaction Mix + Sample + Molecular Water to obtain 20 μL volume. Samples were run in a thermocycler (48-well MJ MiniTM, Bio-rad, Hercules, CA, USA) at 5 min 25 °C, 30 s 42 °C, and 5 s 85 °C. Dilution of the cDNAs was performed 1/50 with water (Molecular Biology Reagent, Sigma-Aldrich, St. Louis, MO, USA). Diluted cDNA (1:50) was used as a template for quantitative PCRs using iTaq Universal SYBR Green Supermix (Bio-Rad, Hercules, CA, USA). Real-time detection of fluorescence emission was performed on a CFX384 Real-Time System (Bio-Rad, Hercules, CA, USA) using the following conditions: denaturalization step 10″ 95 °C followed by annealing and extension 30″ 60 °C. A total of 40 cycles were run. Melt curve was performed, increasing from 65.0 °C to 95.0 °C by 0.5 °C each 5 seconds Plates were edited using the CFX manager version 3.1 software.

Extremely expressed genes across lines at 3 h were chosen as candidate genes to validate transcriptomic experiment using qPCR.

Primers from selected genes were designed using NCBI primer blast tool [112] (Biolegio, Nijmegen, The Netherlands). The sequences of primers used are detailed in Figure S2. The expression of target genes was normalized to the expression level of the Actin and Tubulin genes of *A. thaliana* [113]. The relative expression (RE) of each gene was calculated in comparison to the control treatments (pH 5.9) at each time point. The expression of the target gene relative to the expression of the reference gene was calculated using the 2−ΔΔCt method [114].

### 3.7. Statistical Analysis

For the physiological measurements, normal distribution of the data was confirmed by Levene’s test. Differences between treatments and different demes were analyzed by two-way ANOVA. Post hoc analyses were realized using Tukey’s test. Nutrient mineral data were standardized to avoid bias due to different magnitude orders. Radial plots were used to visualize differences among treatments in each deme. Statistical analyses and plots were performed using JMP13 software (JMP, Version 13. SAS Institute Inc., Cary, NC, USA, 1989–2019).

## Data Availability

Not applicable.

## Supplementary Materials

The following are available online at https://www.mdpi.com/1422-0067/22/3/1226/s1. Figure S1: Growth responses of moderately bicarbonate-tolerant *A. thaliana* deme to different NaHCO_3_ concentrations. (A) Rosette diameter (cm) and (B) root length (cm) of A1_(c+)_ plants grown in hydroponics for 5 weeks. Letters indicate significant differences between treatment (*n* = 9; *p* < 0.05 according to Tukey’s HSD). Figure S2: RNA-seq validation. (A) Primer sequence from eight selected genes used for validation of the RNA-seq expression results. (B) Bar plot from RNA-seq (dark grey) and qPCR (light grey) expression results from the selected genes after 3 h of exposure to bic and pH 5.9. Results are expressed in log_2_ fold change; bars indicate standard error. Figure S3: GO and KEGG pathways of demes comparing alkaline treatment at two time points. Venn diagram of up- and downregulated DEGs comparing bic vs. pH 5.9 and pH 8.3 vs. pH 5.9 treatments after 3 h and 48 h between A1_(c+)_ (A) and T6_(c−)_ (B) demes. (C) Bubble plot indicating significant GO analysis of DEGs in bic vs. pH 5.9 and pH 8.3 vs. pH 5.9 treatments after 3 h and 48 h in A1_(c+)_ and T6_(c−)_ demes. GO were filtered to adjusted *p*-value < 0.05. Scale colors indicate number of DEGs, while bubble size indicates -log of adjusted *p*-value. GO terms were separated into biological function, cellular component, and molecular function. (D) Heatmap plots of KEGG pathway analysis of DEGs in bic vs. pH 5.9 and pH 8.3 vs. pH 5.9 treatments after 3 h and 48 h between A1_(c+)_ and T6_(c−)_ demes. KEGG pathway terms were filtered by *p*-value < 0.05. Scale colors indicate pathway fold enrichment. Supplementary Dataset: Dataset S1: Statistical analysis of physiologic experiments (Figure 1, Figure S1). Dataset S2: Sequencing quality control. Dataset S3: Clean reads mapped into 24,400 genes. Pairwise comparation of alkaline treatments vs. control without filtering. Dataset S4: Slim GO from DEGs (LFC> |1| and adjusted *p*-value < 0.05) in pairwise comparison treatments bic vs. pH 5.9 and pH 8.3 vs. pH 5.9 at different time points (3 h and 48 h) from A1_(c+)_ and T6_(c−)_ demes. Dataset S5: Complete GO from DEGs (LCF> |1| and adjusted *p*-value < 0.05) in pairwise comparison treatments bic vs. pH 5.9 and pH 8.3 vs. pH 5.9 at different time points (3 h and 48 h) from A1_(c+)_ and T6_(c−)_ demes. Dataset S6: KEGG pathway of DEGs (LFC> |1| and *p*-value < 0.05) in pairwise comparison treatments bic vs. pH 5.9 and pH 8.3 vs. pH 5.9 at different time points (3 h and 48 h) from A1_(c+)_ and T6_(c−)_ demes. Dataset S7: Slim GO of upregulated and downregulated DEGs (LFC > |1| and adjusted *p*-value < 0.05) in pairwise comparison bic vs. pH 5.9, from A1_(c+)_ and T6_(c−)_ at 3 h and 48 h. Dataset S8: Complete GO of upregulated and downregulated DEGs (LFC> |1| and adjusted *p*-value < 0.05) in pairwise comparison bic vs. pH 5.9, from A1_(c+)_ and T6_(c−)_ at 3 h and 48 h. Dataset S9: KEGG pathway of upregulated and downregulated DEGs (LFC> |1| and *p*-value < 0.05) in pairwise comparison bic vs. pH 5.9, from A1_(c+)_ and T6_(c−)_ at 3h and 48h. Dataset S10: Top differentially expressed genes at 3 h in A1_(c+)_ and T6_(c−)_. Dataset S11: Protein–protein interaction network functional enrichment analysis output from exclusive genes involved in bic response at 3 h in A1_(c+)_ deme. DEGs filtered (LFC > |1| and adjusted *p*-value < 0.05). Dataset S12: GO and KEGG pathway terms of exclusive genes involved in bic response at 3 h in A1_(c+)_ deme. DEGs filtered (LFC > |1| and adjusted *p*-value < 0.05). Dataset S13: Protein–protein interaction network functional enrichment analysis output of exclusive genes involved in bic 8.3 response at 3 h in T6_(c−)_ deme. DEGs filtered (LFC > |1| and adjusted *p*-value < 0.05). Dataset S14: GO and KEGG pathway terms of exclusive genes involved in bic 8.3 response at 3 h in T6_(c−)_ deme. DEGs filtered (LFC > |1| and adjusted *p*-value < 0.05).

## Abbreviations

ABA	Abscisic acid
ANOVA	Analysis of variance
*A. thaliana*	*Arabidopsis thaliana*
cDNA	Complementary deoxyribonucleic acid
BTP	1,3-Bis[tris(hydroxymethyl)methylamino]propane
DEG	Differentially expressed genes
FDR	Fold discovery rate
GO	Gene Ontology
GS	Glucosinolate
GST	Gluthatione-S-transferase
ICP-OES	Inductively coupled plasma atomic emission spectroscopy
JA	Jasmonic acid
KEGG	Kyoto Encyclopedia of Genes and Genomes
LFC	Log_2_ fold change
MATE	Multidrug and Toxic Compound Extrusion
MES	2-(N-Morpholino)ethanesulfonic acid hydrate, 4-morpholineethanesulfonic acid
NADPH	Nicotinamide adenine dinucleotide phosphate
NCBI	National Center for Biotechnology Information
Adj *p*-value	Adjusted *p*-value
RLK	Receptor-like kinase
RLP	Receptor-like protein
RNA	Ribonucleic acid
ROS	Reactive oxygen species
qPCR	Quantitative Polymerase Chain Reaction
TF	Transcription factor
SA	Salicylic acid
