# Supplementary material for: Transcriptomics Reveals Fast Changes in Salicylate and Jasmonate Signaling Pathways in Shoots of Carbonate-Tolerant Arabidopsis thaliana under Bicarbonate Exposure
Source: Int J Mol Sci. 2021 Jan 27;22(3):1226. doi: 10.3390/ijms22031226 (PMC7865540; doi:10.3390/ijms22031226)
Supplement: Supplementary file 1 [file ijms-22-01226-s001.zip › Supplementary_Figures.pdf]

Supplementary Materials:

Transcriptomics reveals fast changes in salicylate and jasmonate signaling pathways in shoots of carbonate tolerant *Arabidopsis thaliana* under bicarbonate exposure.

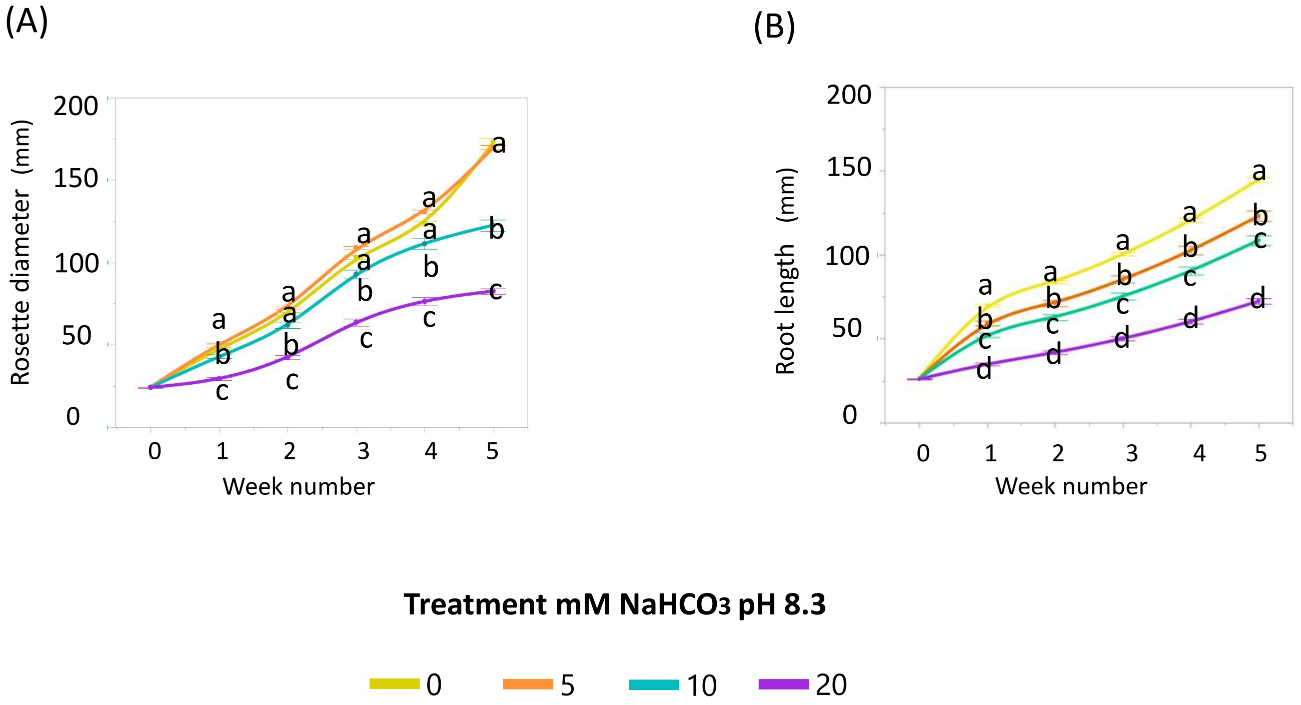

Figure S1: Growth responses of moderately bicarbonate tolerant *A. thaliana* deme to different $NaHCO_3$ concentrations (A) Rosette diameter (cm) and **(B)** root length (cm) of $A1_{(c+)}$ plants grown in hydroponics for 5 weeks. Letters indicate significant differences between treatment (n= 9; $p < 0.05$ according to Tukey's HSD).

.

(A)

Gene Name	TAIR id	LFC_3h_A1(c+)_bic	LFC_3h_T6(c-)_bic	Forward sequence	Reverse sequence
Vacuolar iron transporter (VIT) family protein	AT1G76800	-0.88	-3.13	TTTCCGCAGGTGCGATTGTA	CCTAGCCGATGACCTAACCG
MATE efflux family protein	AT2G04050	-2.23	0.40	CTCTTCACATGCCGAAACATT	TGCCAACCACTTCCCCTTG
Transmembrane protein	AT2G25510	2.50	-1.10	AGGCACAAACGAACCTGGTC	TTATAGCGGCCGTTCGACAA
NAC domain (NAC044)	AT3G01600	-2.08	0.42	GTGGGATTGGTGGCTCTGAA	ATGCCTGCACTGTCTTGTGA
Receptor like protein 39(RLP39)	AT3G24900	1.65	-0.67	TCCTTGGCTTCAAGCTTCTTCT	TATCGCACCACACCCCATTC
Peroxidase superfamily protein	AT4G11290	-1.67	1.04	TTGGTGGACCGACATGGAAC	TGGGCACCGGATAACAAGAC
NADP-malic enzyme 3(NADP-ME3)	AT5G25880	-1.86	0.90	TCGTGTCCGTGATGACATGC	GATTCGATGCCAGTCCGAGT
Late embryogenesis abundant protein (LEA)	AT5G53820	1.07	-2.52	CAGAGCATGAGCTTCAACGC	ATGGTCTCACTCGCACCTTG

(B)

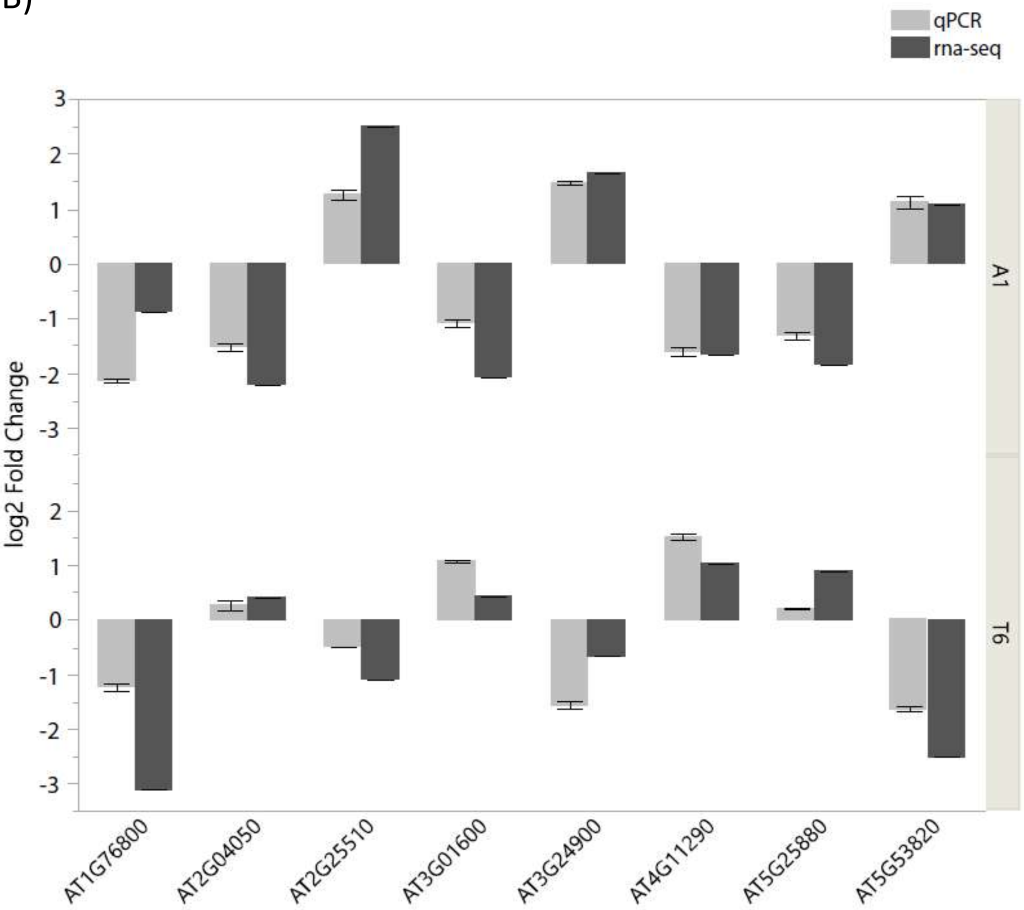

Figure S2: RNA-seq validation. (A) Primer sequence from 8 selected genes used for validation of the RNA-seq expression results. **(B)** Bar plot from RNA-seq (dark grey) and qPCR (light grey) expression results from the selected genes after 3 hours of exposure to bic and $pH_{5.9}$. Results are expressed in log2 fold change ; bars indicate standard error.

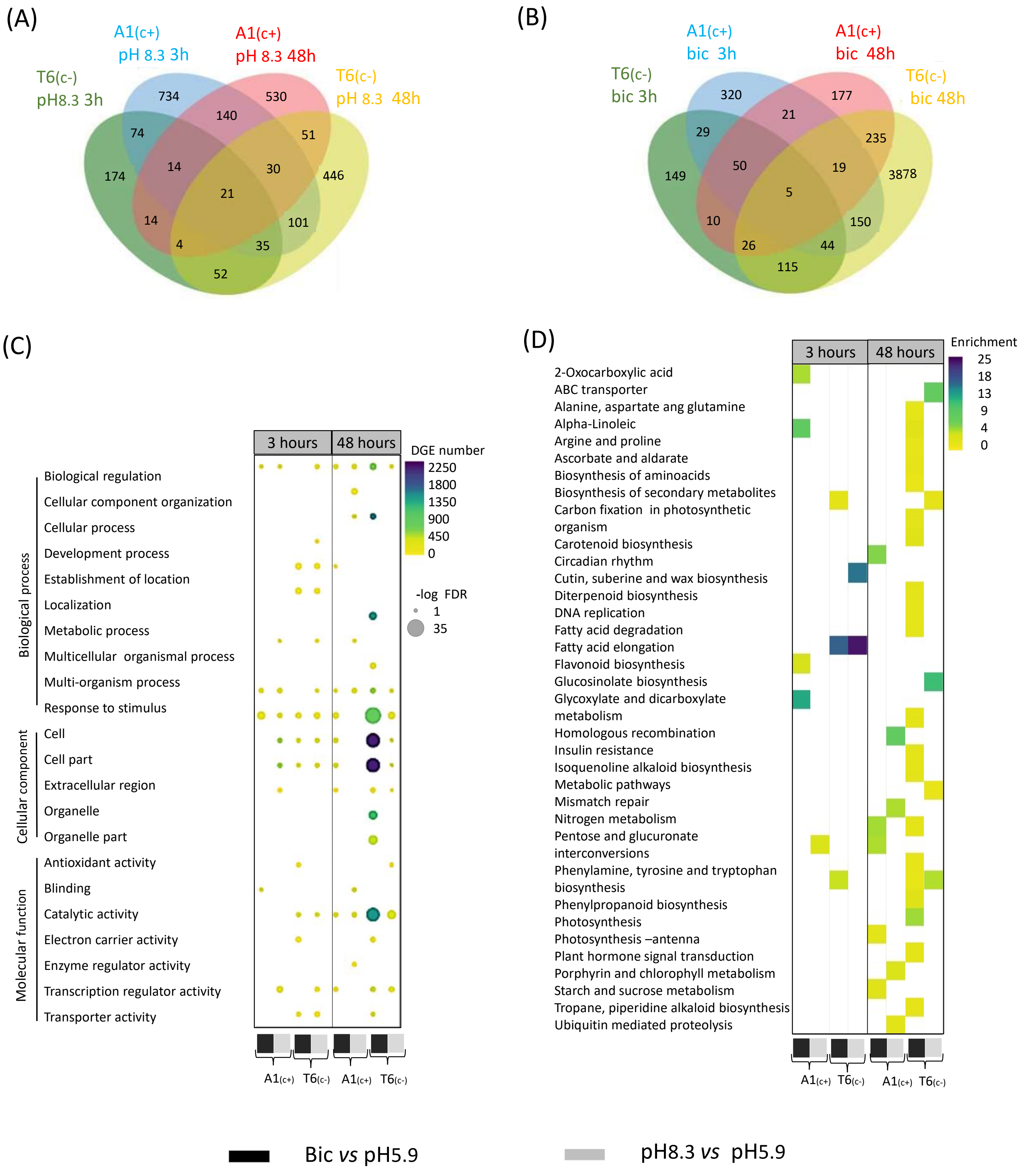

Figure S3: GO and KEEG pathway of demes comparing alkaline treatment at two time points. Venn diagram of up and downregulated DGEs comparing bic *vs* $pH_{5.9}$ and $pH_{8.3}$ *vs* $pH_{5.9}$ treatments after 3 h and 48 h between $A1_{(c+)}$ **(A)** and $T6_{(c-)}$ **(B)** demes. **(C)** Bubble plot indicating significant GO analysis of DGEs in bic *vs* $pH_{5.9}$ and $pH_{8.3}$ *vs* $pH_{5.9}$ treatments after 3 h and 48 h in $A1_{(c+)}$ and $T6_{(c-)}$ demes. GO were filtered to adjusted p-value < 0.05. Scale colors indicate number of DGEs, while bubble size indicates -log of adjusted p-value. GO terms were separated into biological function, cellular component, and molecular function. **(D)** Heatmap plots of KEEG pathway analysis of DGEs in bic *vs* $pH_{5.9}$ and $pH_{8.3}$ *vs* $pH_{5.9}$ treatments after 3 h and 48 h between $A1_{(c+)}$ and $T6_{(c-)}$ demes. KEEG pathway terms were filtered by p-value < 0.05. Scale colors indicate pathway fold enrichment
